# Neighborhood Factors and Fall-Related Injuries among Older Adults Seen by Emergency Medical Service Providers

**DOI:** 10.3390/ijerph14020163

**Published:** 2017-02-08

**Authors:** Sungmin Lee, Chanam Lee, Susan Rodiek

**Affiliations:** 1 Department of Landscape Architecture & Urban Planning, College of Architecture, Texas A&M University, College Station, TX 77843, USA; chanam@tamu.edu; 2 Department of Architecture, College of Architecture, Texas A&M University, College Station, TX 77843, USA; rodiek@tamu.edu

**Keywords:** fall injuries, older adults, aging, elderly, environmental risk factors

## Abstract

Falls are serious health problems among older adults, and are the leading cause of fatal and nonfatal injuries treated by emergency medical services (EMS). Although considerable research has examined the risk factors of falls at the individual level, relatively few studies have addressed the risk factors at the neighborhood level. This study examines the characteristics of neighborhood environments associated with fall injuries reported to EMS providers. A total of 13,163 EMS records from 2011 to 2014 involving adults aged 65 and older in the city of San Antonio (TX, USA) were analyzed at the census tract level (*n* = 264). Negative binomial regression was used to identify significant census tract-based neighborhood environmental variables associated with the count of fall injuries in each census tract. Adjusting for exposure variable and the size of the census tract, neighborhoods with higher residential stability, captured as the percent of those who lived in the same house as the previous year were associated with decreased count of fall injuries. Neighborhoods with higher residential density and having a higher vacancy rate were associated with increased count of fall injuries. The study highlights the importance of stable and safe neighborhoods in reducing fall risks among older adults, which should be considered a prerequisite for promoting age-friendly environments.

## 1. Introduction

Falls are a common and serious health concern as people age. Every year, a fourth of people aged 65 years or older in the United States experience falls, and 20% to 30% of these falls result in nonfatal or fatal injuries [1,2]. In 2014, 2.7 million older adults were treated in emergency rooms for nonfatal falls [3], which typically lead to a decrease in physical activity, quality of life, and social interaction; in the same year, 27,180 adults aged 65 and older in the United States died from serious falls. There are also age differences in risk of falling, with adults aged 85 years or older having greater risk of falling due to the deterioration in their overall health and functional status, compared to those 65–84 years of age [4].

Several risk factors are related to falls through a complex interaction of intrinsic and extrinsic pathways [5]. Intrinsic factors for falls include advanced age, muscle weakness, gait or balance problems, visual deficits, mobility limitations, and cognitive impairments [6]. Extrinsic factors related to falls include medications, assistive devices, and hazardous environments (e.g., an uneven surface and poor lighting) [7]. These risk factors have been determined in previous studies using survey-based fall assessment tools at the individual level. The fall-prevention strategies also tend to focus on individualized approaches, such as exercise, footwear, or individual home safety [8,9,10].

To better understand where, why, and how falls occur, the contexts of residential environments where older adults live and are involved in activities should be taken into account, in addition to attributes of individuals. Studies have shown that physical and social characteristics of the neighborhood not only influence whether older adults remain in their communities or leave, but also influence their physical activities [11,12] and mobility [13]. What is less known is whether and to what extent falls are associated with community-level factors in terms of demographic, socioeconomic status, and physical environments?

Meanwhile, sociologists and urban planners have long identified the importance of neighborhood context as a structural factor that influences individual lives and access to opportunities [14]. Concentrated disadvantages, including residential instability, social segregation, and poverty have been investigated from socio-ecological perspectives and shown to influence various social outcomes such as fear of crime and child development [15,16]. Increasingly, epidemiologists and public health experts have also become aware of geographical inequality and the importance of places on health [17,18]. In recent research, neighborhood characteristics such as family stability, crime, unemployment, and housing conditions have been shown to be associated with overall mortality, disease prevalence, health behaviors, and mental health outcomes [19,20]. A growing body of research examining the spatial clustering of mortality, homicide, and accidental injury has enabled researchers and practitioners to identify geographic hot spots linked with a wide range of health outcomes, enriching the discussions on the neighborhood-level risk factors [21,22].

Injuries among older adults have plausible associations with the neighborhood environment because most age-related falls occur in residential areas. Due to the combination of increased physical and psychological vulnerability, mobility limitations, and changing patterns of spatial use with age, neighborhood environments play an important role in maintaining health and mobility among older adults [13,23]. A decrease in physical and cognitive capacity such as visual, balance, and cognitive impairment can result in older adults failing to cope with hazardous environments, and can lead to nonfatal and fatal injuries [24]. Neighborhoods with low socioeconomic status and high socio-physical disorder such as vacant buildings and crime tend to have poor maintenance conditions [25], which may be associated with fall risks in vulnerable older adults with visual or balance impairments.

Several conceptual models describe neighborhood context as a risk factor associated with injury-related outcomes. The Haddon matrix, a commonly used tool in the injury prevention field, has been employed to identify factors related to environmental attributes as well as personal and agent attributes [26]. The social determinants of health model highlights the environmental factors (e.g., stressful living conditions) and structural contexts (e.g., housing policies) that influence individual socioeconomic position and health outcomes [27,28]. Such frameworks of neighborhood effects help explain the association between geographical measures of neighborhood contexts (e.g., social class, socioeconomic status, accessibility to community resources) and the clustering of injury outcomes [29]. For example, Baker and his colleagues (1987) found geographical variations in motor vehicle mortality rates associated with population density and capital income at the county level [30]. Some studies on falls found regional variations in demographics of residents hospitalized for falls, and identified high-incidence areas at the county level [31]. However, these studies did not identify the effects of neighborhood context on the clustering of falls. Determinants of fall clustering have not been well explored in terms of neighborhood characteristics, such as demographic, socioeconomic status, residential stability, socio-physical disorder, and land use.

Given the limited availability of spatially-based fall data and the difficulties in identifying the location and health information of fall injury patients, this research used fall data reported by emergency medical services (EMS). EMS provides identifiable, objective, and representative data on fall-related injuries. The aims of this study are to: (a) examine the spatial inequality of fall-related injuries, and (b) estimate the effects of neighborhood contexts on fall injuries among older adults by different age groups (65–84 versus 85 years and older) at the census tract level. Although the census tract is still an aggregated geographic unit of analysis, it is much more fine-grained than those used in previous studies (e.g., County) and tracts have often been used as an acceptable neighborhood unit for large population studies lacking individual-level data [32]. A central hypothesis that underlies these aims is that fall-related injuries do not occur evenly across areas. We also hypothesized that certain neighborhood contexts (e.g., socioeconomic status, rates of frequent residential mobility, and socio-physical disorders) are associated with the clustering of fall injuries.

## 2. Methods

### 2.1. Data Source

This study used data from several sources. We used fall-related injuries data (2011–2014) reported by the EMS and the San Antonio Fire Department (SAFD) in the city of San Antonio (TX, USA). For the purpose of this study, we restricted the analysis to fall events of people aged 65 and older with a mean age of 80.6 reported from 2011 to 2014 (*n* = 13,178). For the neighborhood variables, we used the 2013 American Community Survey (ACS) data from the U.S. Census, the parcel-level land use data from the Bexar County Tax Appraisal District, and the property crime incidence data from the San Antonio Police Department. Geographic Information System (GIS) was used to generate the residential land use from the parcel data. The ACS data provided information about the socio-demographic and -economic status (i.e., median household income, number of aging residents), residential stability (i.e., owner-occupied housing rate, and the rate of residents living in the same house as the previous year), household structure, housing condition, dwelling types, and vacancy rate. The property crime data included all reported property crime incidents from 2011 to 2014. The spatial unit of analysis was the census tract; we used all 264 census tracts within the city of San Antonio for the neighborhood-level analysis after excluding census tracts that had missing data or <500 residents.

### 2.2. Dependent Variable 

To ascertain whether neighborhood context was associated with the count of fall-related injuries at the neighborhood-level, we used the total count of fall injuries by older adult groups stratified by age (65–84 years and ≥85 years) during the period of 2011 through 2014 at the census tract level as the outcome. Initially, the fall data reported by EMS was injury-entailed events which are different from general falls that broadly encompass light slips or trips without injuries. Since this dataset was pulled from the medical reports, it shows the number of actual patients who required medical treatment when EMS providers arrived at the scene. Thus, the count of neighborhood falls used in this study is the aggregated count of fall injuries occurred among different age groups collected by EMS providers at each census tract.

The fall location data contained the street name and zip code without the street number, which generated incompletely geocoded cases. Although the dataset did not allow us to identify exact point-based geocoded locations, it enabled us to assign cases to street-based geocoded address lines depending on the street name and zip code. Those street-based geocoded address lines were then assigned to the corresponding census tract(s). Table 1 showed the number and percentages of fall injuries in the City of San Antonio by age category and geocoding status. More than half (*n* = 8720; 66.25%) of the street-based geocoded lines with fall incident(s) were contained within a single census tract, while the rest ran across two or more census tracts. These two types of address lines are not distributed at random, because most local and residential streets are short while arterial/collector roads and highways often travel across multiple census tracts [33].

For the incompletely geocoded cases (*n* = 4443; 33.75%) we used a geographical imputation method to assign street-based geocoded cases to the overlapping census tracts based on the proportion of the street overlapping each tract and the aging population accounted for by each census tract. Although several geographic imputation methods have been used to reduce non-geocoded error, those imputation methods were conducted to assign non-geocoded cases to census tracts based on ZIP code centroid [34,35]. Since street-based geocoded line within ZIP code is more accurate than ZIP code centroid, we adopted this imputation strategy after considering population density of the census tract, consistent with previous studies [34]. For example, if 0.3 miles of a 1-mile long geocoded line with a fall incident is located in census tract A that has 90 older (aged 65+) residents and 0.7 miles in census tract B having 140 older residents, the weighted A (90/0.3) to B (140/0.7) ratio is 3 to 2, resulting in 0.6 and 0.4 incidents to be assigned to tract A and B, respectively.

### 2.3. Independent Variables

To analyze the neighborhood environmental factors and other neighborhood safety conditions, eleven independent variables were included, classified into five groups: demographic and socioeconomic status, residential stability, household structure, housing condition, dwelling type, and socio-physical disorder. First, as part of demographic and socioeconomic status, we included net population density, median household income, and poverty rate in older adults at the census tract level. Residential density is typically associated with housing conditions and residential dwelling types, and injurious falls among older adults are disproportionately associated with home hazards. Households with lower incomes and houses in low income neighborhoods tend to lack fall prevention devices (e.g., stair handrails, grab bars) and have poor and hazardous conditions (e.g., poor stair design and dim lighting) for older residents [36]. Second, residential stability was identified by the degree to which neighborhoods were stable, and included the proportion of owner-occupied housing units and the proportion of the residents that had lived in the same house for a year [37]. Given the negative impact of unsustainable forms of residence on health and well-being among older adults [38], housing stability may be an important consideration in understanding the risks in falling at the neighborhood level. Third, we added household structure because older adults living alone tend to have greater fear of falling and fall-related injuries compared to those living with others [39]. In addition, several studies have indicated that older buildings have lower levels of maintenance, which in turn may lead to fires, collapse, or other injuries [40]. Since injurious falls were associated with types of living environment, we included dwelling type as an independent variable. Finally, to capture neighborhood socio-physical disorders, we included vacancy rate and property crime [25] that occurred between 2011 and 2014, a period that was consistent with the outcome data. These socio-physical disorders are well known to be associated with decreased physical activity levels as well as being proxy measures of poorly maintained neighborhood and housing conditions. We excluded pedestrian infrastructure or street condition associated with outdoor falls, because most EMS-based fall injuries occurred within the home.

### 2.4. Control Variables

Since we targeted fall incidents occurring among older adult groups, we added number of residents stratified by age as an exposure variable to account for the exposure issue, as census tracts having more older adults will likely have more fall incidents by older adults, regardless of the neighborhood conditions. Also, the spatial size of census tracts varies, with larger census tracts being generally located in the periphery of metropolitan areas with low population density [41]. To control for the statistical effects of different census tract sizes on the possibilities of fall incidents, we included census tract acreage as a control variable. To help mitigate the previously reported limitation with the fall location data missing the street number, we further included an incompleteness rate variable, calculated as the number of partially geocoded falls using the proportional allocation method out of total fall counts within the census tract.

### 2.5. Analytical Approach

Due to the skewed distribution and overdispersion of the dependent variable of total fall injuries in the census tract, we used a negative binomial model according to age categories (model 1: age 65+, model 2: age 65–84, and model 3: age 85+). Each of the independent variables was tested by adding them one at a time after being controlled for confounding variables in each model. Independent variables with a *p*-value < 0.05 were then considered for the final multivariate analysis after being checked for multicollinearity. The analysis was conducted with STATA IC 12.0 (Stata Corp., College Station, TX, USA). The incidence rate ratio (IRR) and 95% confidence intervals were reported. The IRR typically reports the estimated rate ratio of the incidence occurrence for a one unit increase in the independent variable [42]. Negative binomial regression is adaptable for modeling count variables and can easily generate the estimates of prevalence. In this study, we used fall counts instead of fall rates (total falls divided by population or area) as the outcome variable due to ease of interpretation.

## 3. Results

### 3.1. Prevalence of Neighborhood Falls and Characteristics of Neighborhood Contexts

From 2011 through 2014, a total of 26,901 fall-related injuries were reported by EMS agencies in San Antonio. About half (*n* = 13,163, 48.9%) of those incidents involved older adults aged 65+. Among them, 8237 (62.6%) and 4926 (37.4%) incidents involved adults aged 65–84 and aged 85 or older respectively. Table 2 illustrates univariate descriptive statistics for all variables used in the analysis at the census tract level.

**Figure 1:**
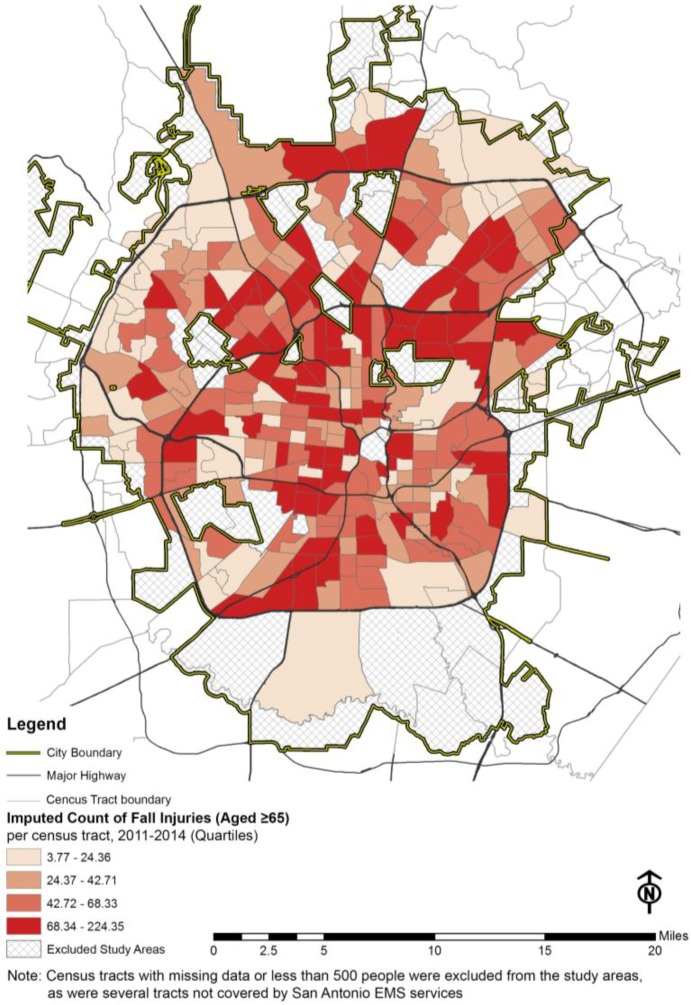
EMS recorded fall-related injuries (aged ≥ 65) per census tract, city of San Antonio, 2011–2014.

The count of fall injuries among people aged 65 and older between 2011 and 2014 within the census tracts was spatially over-dispersed (mean = 51.50, standard deviation = 35.76), which meant that the fall events were not distributed normally or equally across the city. In other words, certain contextual factors were contributing to the geographically inequitable distribution of fall incidents. The census tract level geographic distribution of fall incidents (counts of falls among the residents aged 65 and older) is presented in Figure 1.

### 3.2. Neighborhood Contexts Associated with Counts of Falls at the Neighborhood Level

Table 3 shows the partially adjusted model for the relationship between neighborhood contexts and fall-related injuries by age groups. The factors influencing the fall injuries that occurred among the age 65–84 group slightly differed from those affecting the fall injuries that occurred among the age 85+ group. After adjusting for exposure and confounding variables, median household income and percent of household living alone were significantly associated with the count of fall injuries in the aged 65+ group model 1 and aged 65–84 group model 2, but not in the 85+ group model 3. However, percent of older adults below poverty level was highly and significantly associated with the count of fall injuries in the aged 85+ group model only. Other variables, such as percent of owner-occupied, percent of residence 1 year and over, percent of single- and multi- family units, percent of vacant housing units, and property crime, were strongly associated with count of fall injuries in 65+ group model, 65–84 group model, and 85+ group model independently.

In the 65+ group model, the areas with high residential stability were significantly associated with a decreased count of fall injuries. The percent of those who lived in the same house as the previous year (IRR = 0.980, 95% CI = 0.975–0.985, *p* < 0.01) and the percent owner-occupied housing (IRR = 0.972, 95% CI = 0.965–0.979, *p* < 0.01) were shown to be associated with a decreased count of fall injuries. Neighborhoods with a higher percent of older adults below poverty level (IRR = 1.308, 95% CI = 0.984–1.739, *p* < 0.05), a higher percent of household living alone (IRR = 1.006, 95% CI = 1.000–1.010, *p* < 0.01), a higher percent of vacant housing units (IRR = 1.033, 95% CI = 1.024–1.043, *p* < 0.01), and a higher property crime incidence (IRR = 1.077, 95% CI = 1.036–1.119, *p* < 0.01) were associated with higher fall incidence. The magnitude of influence that property crime had on total falls, however, was not strong, with each additional property crime in 1000 increasing the count of fall injuries by only 7.7%. In addition to residential stability and neighborhood quality, neighborhoods with lower percent of single-family units (IRR = 0.992, 95% CI = 0.990–0.994, *p* < 0.01) and higher percent of multi-family units (IRR = 1.008, 95% CI = 1.007–1.010, *p* < 0.01) were associated with higher fall incidence.

### 3.3. Multivariate Analysis of Risk Factors for Counts of Fall-Related Injuries at the Neighborhood Level

Table 4 displays adjusted incidence rate ratios from the multivariate analysis according to different age group model. Percent older adults below poverty level, percent owner-occupied, percent single-family units, and percent vacant housing units were dropped from further analyses due to multicollinearity.

Also, percent older housing was excluded from the multivariable-adjusted analysis because of statistically low significance (*p* > 0.05). In the aged 65+ group model 1, three variables were significantly associated with the count of fall injuries after controlling all other significant variables. Increased residential density was significantly associated with an increased count of fall injuries. This may be because neighborhoods with low residential density tend to have better housing conditions, compared to areas with high residential density. For residential stability, each one percentage point increase in the population 1 year and over by length of residence was associated with a 1.1% decrease in the count of fall injuries (IRR = 0.989, 95% CI: 0.983–0.995, *p* < 0.01), while holding all other variables in the model constant. The housing vacancy rate (IRR = 1.022, 95% CI: 1.012–1.032, *p* < 0.01) also remained significant. For each one percentage point increase in vacancy rate, the count of fall injuries that occurred among those aged 65 and over increased by 2.2%. Finally, socio-physical disorder was more strongly associated with the fall injuries that occurred among those aged 85+ (% vacant housing units: IRR = 1.032, 95% CI: 1.013–1.052, *p* < 0.01), compared to those aged 65–84 (% vacant housing units: IRR = 1.015, 95% CI: 1.005–1.024 *p* < 0.01) in the comparison between model 2 and model 3.

## 4. Discussion

This paper explores characteristics of fall-vulnerable areas in terms of demographic and socio-economic status, residential stability, household structure/housing condition, dwelling type, and socio-physical disorder. The findings indicate that low income areas have more fall injuries than high income areas, because housing in low income areas tends to have poor maintenance, lack of opportunities to participate in fall-prevention programs, and lack of home-safety devices for seniors. The finding that the areas with high residential stability were negatively associated with fall-related injuries suggests the importance of stable neighborhood environments in preventing fall injuries among older adults. Generally, residential stability at the neighborhood level is known to be associated with neighborhood quality and psychosocial stressors [27,43]. Previous studies showed that length of residence and housing ownership, as measures of residential stability, may also influence the level of familiarity with one’s home and surroundings [44,45]. In other words, low residential stability due to decreased financial resources after retirement, and desire to be closer to family, health care, or amenities, may contribute to an increased exposure to unfamiliar residence or neighborhoods among older individuals, and could possibly increase the likelihood of injuries from falls [46].

In addition to residential stability, the results also indicated that improving neighborhood security and reducing socio-physical disorder could help prevent fall injuries. High vacancy rates, daily exposure to threatening environments, fear of property crime, and anxiety about neighborhood crime could make older adults avoid going outside, which could result in reduced physical and social activities [47,48]. Such reduced activities could lead to mobility impairments and unintentional falls inside the home [49]. Despite the lack of studies directly examining the relationship between socio-physical disorder and falls, several previous studies have supported the idea that threatening and hazardous environments characterized by crime, danger, and incivility would be associated with other health-related outcomes such as increased fear, physical inactivity, and decreased walking [25,50].

Findings from the multivariate model suggest that urgent attention to fall prevention is needed in areas with a large population of older adults, short residential duration, and high vacancy rates. People living in such neighborhoods are prone to lack sufficient social ties or are vulnerable to the risk of crimes as well as injuries from falls [15]. Thus neighborhood interventions to promote stability, decrease mobility and displacement, and mitigate vacancy rates may serve an important role in preventing falls among older adults at the neighborhood level. For example, a rental stability program could offer a senior tenant an opportunity for longer lease terms [51], and might be helpful in preventing or reducing fall injuries. Also, strategies for stronger markets, such as foreclosure prevention programs and rehabilitation for sale, could assist owner and renter occupants to remain in their homes [52] and therefore contribute to reducing the risk of fall incidents among older adults.

This study also found that fall injuries among the 85+ group tended to be less influenced by household structure (% living alone), compared to fall injuries among the 65–84 age group at the neighborhood level. However, residential stability (e.g., housing ownership and length of residence) and socio-physical disorder (e.g., vacant housing units and property crime) were both associated with fall injuries among the 65–84 age group and the 85+ age group. Given the fact that risk of falling in those 85 years and older appears to be greater than in those 65–84 years of age at the individual level, the intrinsic factors of fall injuries, such as health status, mobility limitations, and gait or balance would be more associated with oldest-old group compared to young- and middle- old group [4]. However, neighborhood- based extrinsic factors or social determinants of fall injuries are both highly related to those aged 65–84 and 85+, while different neighborhood intervention strategies would be necessary in areas where fall injuries occurred among those aged 65–84 and those 85+ years of age.

While this study has provided insights into potential preventive strategies to reduce neighborhood fall-related injuries, some limitations should be acknowledged. This study failed to incorporate individual-level variables such as health conditions, balance impairments, cognitive problems, and other health factors that would be important determinants of individual fall incidents. The Health Insurance Portability and Accountability Act (HIPPA) privacy rule protects access to individual medical records [53]. Further studies could examine within- and between- neighborhood variability in fall incidents employing multilevel modeling approaches. This study also relied on a cross-sectional approach that failed to draw causal inferences between variables. A longitudinal study should be undertaken to determine the relationship between the duration of where people live, and fall incidence.

The EMS-based fall data have both strengths and limitations. First, the population-based data enable researchers to estimate the relationship between neighborhood environments and fall-related injuries. Compared to a self-surveyed measurement of falls that relies on sample participants, falls collected by EMS providers were more reliable and systematic, providing fairly accurate location data. However, the data of falls seen by EMS providers were somewhat limited in locational accuracy to be precisely geocoded at this time, as the released address data lacked the street number. Thus, we were not able to use a smaller unit of analysis and had to use the census tract as the spatial unit.

Moreover, it was not possible to distinguish between indoor and outdoor falls in our analysis. In the EMS data at the county and state level, the locations of fall incidents were documented, but the location information was not released at the census tract level. People who experienced falls in homes versus outdoors such as on sidewalks or streets have different risk profiles: indoor fallers tend to be female, have worse health status, and have balance impairment, while outdoor fallers are more likely to be physically active [54]. Moreover, the outdoor neighborhood environment, such as walkability, street connectivity, and other street conditions are relevant to outdoor falls only [55]. Not surprisingly, most fall incidents seen by EMS providers occurred in homes, according to the Texas EMS/Trauma Registry. City-specific EMS-based fall data are limited but the regional data combining the City of San Antonio and Bexar County showed only 3.06% of the total falls among people aged ≥65 years occurred in streets or sidewalks. Further research is needed to analyze the relationships between outdoor built environments and outdoor falls, due to the scarcity of available information, compared to the literature on indoor and personal risk factors for falls. Relying on the census tracts as a proxy of neighborhoods is another limitation. Using census tracts may not fully reflect a meaningful definition of neighborhood, and the variability of fall incidents within the census tract could not be examined.

Despite these limitations, the study provides information about “fall hot spots” where falls among older adults are spatially concentrated. Our identification of fall hot spots suggests that EMS providers, gerontologists, public health experts, and policy makers can further explore why fall incidents occur and are concentrated in certain places, making it possible to develop effective fall prevention strategies to mitigate falls and subsequent injuries. For example, with the information on a fall hot spot map, fire departments and EMS providers can prepare medical treatments and provide ambulances to respond immediately and adequately to the fall injury patients. Transportation and urban planners can help find ways to minimize the arrival time of ambulances and increase accessibility to hospitals in areas prone to fall incidence. Also, policy makers could use this map to locate EMS or medical facilities appropriately, considering fall incidence rates in addition to other population and health-related conditions.

## 5. Conclusions 

Given the high prevalence and healthcare costs related to injuries from falls, it is important to comprehensively approach fall prevention at both the individual and neighborhood levels. The findings from this study suggest that multifaceted community interventions to create stable and safe neighborhoods could play a significant role in preventing fall-related injuries. These findings help identify fall-related risk factors in the neighborhood environment; this can be especially beneficial to the health and quality of life of older adults, who are at the highest risk of falling and being injured from falls. These findings can also help policy-makers, healthcare/EMS providers, and urban planners consider fall-related injuries in their efforts toward promoting healthy aging and creating age-friendly neighborhoods.

## Figures and Tables

**Table 1:** Number and percentages of fall injuries in the City of San Antonio, by age category and geocoded status, 2011–2014.

Count of Fall Injuries	(Aged 65+) *n* = 13,163		(Aged 65–84) *n* = 8237		(Aged 85+) *n* = 4926	
	*n*	%	*n*	%	*n*	%
Completely geocoded cases	8720	66.25	5080	61.67	3157	64.09
Incompletely geocoded cases	4443	33.75	3157	38.33	1769	35.91

**Table 2:** Descriptive statistics of study variables for 264 census tracts in San Antonio, TX.

Variable	Definition	Mean	SD	Min	Max
Dependent variable					
Fall injuries (aged 65+)	Imputed total fall count among residents by age groups between 2011–2014	51.50	35.76	3.77	224.35
Fall injuries (aged 65–84)		32.68	19.85	3.61	112.08
Fall injuries (aged 85+)		18.81	18.46	0.00	116.20
Confounding variable					
Incompleteness rate (aged 65+)	Count of incompletely geocoded fall cases/total imputed fall counts within census tract by age groups	0.37	0.27	0.00	1.00
Incompleteness rate (aged 65–84)		0.38	0.27	0.00	1.00
Incompleteness rate (aged 85+)		0.36	0.29	0.00	1.00
Population aged 65+	Number of population by age groups (Exposure variable)	504.98	258.42	62.00	1401.00
Population aged 65–84		437.43	217.75	62.00	1305.00
Population aged 85+		67.55	66.84	0.00	448.00
Areas (acres/1000)	Size of each census tract (acres/1000)	0.87	1.00	0.18	11.46
Demographic and socioeconomic status					
Net population density	Total population/residential land acreage	17.74	7.79	2.23	78.31
Median household income ($/10,000)	Median household income ($/10,000)	4.67	2.51	0.98	18.59
% older adults below poverty level	Residents aged 65+ living at poverty level/total residents aged 65+ × 100	13.90	11.65	0.00	57.60
Residential stability					
% owner-occupied	Owned housing units/total housing units × 100	20.00	7.00	2.24	39.02
% residence 1 year and over	Residents living in the same house 1 year ago/total residents × 100	81.10	10.56	34.75	97.97
Household structure and housing condition					
Percent living alone	Living alone households/family households × 100	36.52	13.96	3.21	85.60
% older housing	Housing units built before 1950/total housing units × 100	21.39	31.71	0.00	94.17
Dwelling type					
% single-family units	Single-family housing units/total housing units × 100	78.35	26.93	1.57	120.80
% multi-family units	Multi-family housing units/total housing units × 100	31.78	29.53	0.00	126.89
Socio-physical disorder					
% vacant housing units	Vacant housing units/total housing units × 100	10.28	5.93	0.00	33.08
Property crime (*n*/1000)	Total property crime count between 2011–2014 (*n*/1000)	2.63	1.65	0.23	11.82

**Table 3:** Partially adjusted analysis: Neighborhood context and count of fall-related injuries at the census tract level (*n* = 264).

Variable	Model 1		Model 2		Model 3	
	Count of Fall Injuries (Aged 65+) ^a^		Count of Fall Injuries (Aged 65–84) ^a^		Count of Fall Injuries (Aged 85+) ^b^	
	IRR (95% CI)	*p*-Value	IRR (95% CI)	*p*-Value	IRR (95% CI)	*p*-Value
Demographic and socioeconomic status						
Net population density	1.021 ** (1.014–1.029)	<0.001	1.021 ** (1.014–1.028)	<0.001	1.018 ** (1.005–1.031)	0.006
Median household Income ($/1000)	0.947 ** (0.923–0.972)	<0.001	0.941 ** (0.918–0.965)	<0.001	0.963 (0.914–1.015)	0.159
% older adults below poverty level	1.308 * (0.984–1.739)	0.020	1.257 (0.962–1.641)	0.094	2.077 * (1.045–4.129)	0.037
Residential stability						
% owner-occupied	0.972 ** (0.965–0.979)	<0.001	0.972 ** (0.966–0.979)	<0.001	0.977 ** (0.963–0.99)	0.001
% residence 1 year and over	0.980 ** (0.975–0.985)	<0.001	0.984 ** (0.979–0.988)	<0.001	0.978 ** (0.968–0.987)	<0.001
Household structure and housing condition						
% living alone	1.006 ** (1.000–1.010)	0.007	1.007 ** (1.002–1.011)	0.002	1.003 (0.995–1.011)	0.449
% older housing	1.000 (0.999–1.003)	0.486	1.001 (0.999–1.003)	0.305	1.001 (0.997–1.005)	0.615
Dwelling type						
% single-family units	0.992 ** (0.990–0.994)	<0.001	0.993 ** (0.991–0.995)	<0.001	0.992 ** (0.988–0.995)	<0.001
% multi-family units	1.008 ** (1.007–1.010)	<0.001	1.007 ** (1.005–1.008)	<0.001	1.009 ** (1.006–1.012)	<0.001
Socio-physical disorder						
% Vacant housing units	1.033 ** (1.024–1.043)	<0.001	1.026 ** (1.016–1.035)	<0.001	1.042 ** (1.024–1.06)	<0.001
Property crime (*n*/1000)	1.077 ** (1.036–1.119)	<0.001	1.073 ** (1.035–1.112)	<0.001	1.075 * (1.008–1.147)	0.029

Notes: ** *p* < 0.01, * 0.01 ≤ *p* < 0.05; Adjusted by negative binomial model for confounding variables: incomplete rate, population (older adults) stratified by age (exposure variable), areas; **^a^** *n* = 264; and **^b^** *n* = 239.

**Table 4:** Multivariable-adjusted analysis: Neighborhood context and count of fall-related injuries at the census tract level (*n* = 264).

Variable	Model 1		Model 2		Model 3	
	Count of Fall Injuries (Aged 65+) ^a^		Count of Fall Injuries (Aged 65–84) ^a^		Count of Fall Injuries (Aged 85+) ^b^	
	IRR (95% CI)	*p*-Value	IRR (95% CI)	*p*-Value	IRR (95% CI)	*p*-Value
Incompleteness rate (aged 65+)	0.852 (0.688–1.055)	0.779	-	-	-	-
Incompleteness rate (aged 65–84)	-	-	0.862 (0.699–1.062)	0.163	-	-
Incompleteness rate (aged 85+)	-	-	-	-	0.611 ** (0.419–0.891)	0.010
Population aged 65+	(Exposure)	-	-	-	-	-
Population aged 65–84	-	-	(Exposure)	-	-	-
Population aged 85+	-	-	-	-	(Exposure)	-
Area (acres/1000)	0.959 (0.898–1.023)	0.202	0.995 (0.935–1.059)	0.880	0.904 (0.797–1.026)	0.117
Net population density	1.010 * (1.002–1.019)	0.020	1.012 ** (1.004–1.021)	0.003	1.006 (0.99–1.022)	0.481
Median household Income ($/1000)	0.993 (0.959–1.029)	0.708	0.976 (0.943–1.009)	0.153	1.019 (0.943–1.101)	0.634
% residence 1 year and over	0.989 ** (0.983–0.995)	<0.001	0.993 * (0.987–0.998)	0.011	0.986 * (0.974–0.998)	0.022
% living alone	0.996 (0.990–1.002)	0.198	0.996 (0.991–1.002)	0.169	0.996 (0.985–1.007)	0.460
% Vacant housing units	1.022 ** (1.012–1.032)	<0.001	1.015 ** (1.005–1.024)	0.002	1.032 ** (1.013–1.052)	0.001
Property crime (*n*/1000)	1.035 (1.000–1.071)	0.050	1.032 (0.999–1.067)	0.056	1.059 (0.995–1.127)	0.070

Notes: ** *p* < 0.01, * 0.01 ≤ *p* < 0.05, *n* = 264; Model 1 (LR Chi2 = 1458.81; *p*-value < 0.001); Model 2 (LR Chi2 = 552.95; *p*-value < 0.001); Model 3 (LR Chi2 = 1454.81; *p*-value < 0.001); **^a^** *n* = 264; and **^b^** *n* = 239.
